# Predicting the HIV/AIDS epidemic and measuring the effect of AIDS Conquering Project in Guangxi Zhuang Autonomous Region

**DOI:** 10.1371/journal.pone.0270525

**Published:** 2022-07-01

**Authors:** Shizhao Ma, Yi Chen, Xiulan Lai, Guanghua Lan, Yuhua Ruan, Zhiyong Shen, Qiuying Zhu, Shuai Tang

**Affiliations:** 1 Institute for Mathematical Sciences, Renmin University of China, Beijing, China; 2 Guangxi Key Laboratory of Major Infectious Disease Prevention and Control and Biosafety Emergency Response, Guangxi Center for Disease Control and Prevention, Nanning, China; 3 State Key Laboratory of Infectious Disease Prevention and Control (SKLID), Chinese Center for Disease Control and Prevention (China CDC), Collaborative Innovation Center for Diagnosis and Treatment of Infectious Diseases, Beijing, China; Padjadjaran University: Universitas Padjadjaran, INDONESIA

## Abstract

To control the HIV/AIDS epidemics in Guangxi Zhuang Autonomous Region in China, Guangxi government launched the 5-year Guangxi AIDS Conquering Project (GACP, Phase I: 2010-2014, Phase II: 2015-2020). In the project, three measures are implemented, such as great improvements of the coverage of HIV/AIDS education, promotion of HIV voluntary counseling and testing, and enhancement of antiretroviral treatment. In this paper, we explore the effects of the three measures of GACP by construction of a Susceptible-Infected-Diagnosed-Treated population compartments model and via evaluation of the basic reproduction number derived from the model. A computational framework is developed for estimating the model parameters based on the HIV surveillance data, with application of the Markov-Chain Monte-Carlo method and Nonlinear Least Squares method. By estimating the new infections and evaluating the basic reproduction number, we find that the implementation of the three measures of GACP has a significant effect on controlling the rise of HIV/AIDS cases and the epidemic trend. Compared with HIV voluntary counseling and testing, strengthening HIV/AIDS education and expanding the coverage of antiretroviral treatment show a greater impact on HIV/AIDS epidemic control, which provides a reference project for other provinces with a similar epidemic situation in Guangxi Zhuang Autonomous Region. At the same time, our research fills the current research gap for the evaluation of large-scale AIDS prevention and control projects in developing areas.

## Introduction

Acquired immune deficiency syndrome (AIDS) is a disease caused by human immunodeficiency virus (HIV) infection [1–3]. It is a world-widely spreading infectious disease. The epidemic is especially not optimistic in underdeveloped areas of the world. So far, the spread of HIV has not been effectively controlled [4]. By the end of 2020, 36.3 million people had died of AIDS worldwide [5], which is one of the most deadly epidemics in human history. Since the first case of AIDS appeared in June 1985, AIDS has been rapidly spreading in Chinese mainland [6].

Since 2003, the Chinese government has implemented the policy of Four Free and One Care (the National Program), with the implements of measures of providing free antiviral drugs to HIV/AIDS patients with financial difficulties, providing free HIV testing to AIDS patients, providing free schooling for AIDS orphans, providing free treatment for AIDS pregnant people, and providing governmental assistance to AIDS patients with financial difficulties [7]. Since the implementation of these policies in 2003, more and more AIDS patients have received formal HIV testing and antiretroviral treatment (ART), and the coverage of antiretroviral treatment has been expanding in China. This policy has greatly reduced the death rate of HIV/AIDS, which is greatly helpful to HIV/AIDS prevention and control in China.

By the end of 2020, there were 1.053 million people with HIV and 351,000 cumulative reported deaths in China, of which 978,138 received ART. The proportion of HIV-infected people receiving ART was 92.9% and the success rate of treatment was 93.5%. In the past decade, the Chinese government has conducted large-scale AIDS screening and testing and the total number of HIV testing has increased from 55.6 million in 2009 to more than 240 million in 2020 [8]. Significant progress has been made in HIV/AIDS prevention and control in the Chinese mainland, with the overall epidemic kept at a low epidemic level [9].

Guangxi Zhuang Autonomous Region is located on a major heroin trafficking route, and the HIV epidemic in Guangxi Zhuang Autonomous Region was initiated in the 1990s by injection drug users. Since then sexually transmitted HIV infections have increased constantly. According to the official data of China Ministry of Health [10], Guangxi Zhuang Autonomous Region was the province with the second highest number of reported HIV/AIDS cases in all provinces of the Chinese mainland by 2011. In response to the severe epidemic of HIV, the Guangxi government launched the Five-year Guangxi AIDS Conquering Project (GACP) in 2010 [11]. On the basis of the national policy of Four Free and One Care, GACP further strengthened HIV prevention education, HIV voluntary counseling and testing, surveillance, intervention, medical care and antiretroviral treatment in Guangxi Zhuang Autonomous Region, creating a favorable environment for HIV/AIDS prevention and control.

From 2010 to 2020, a Two-cycle GACP project had been implemented in Guangxi Zhuang Autonomous Region, that is, Phase I: 2010–2014, and Phase II: 2015–2020. Evaluating the impact of GACP on the HIV/AIDS epidemic in Guangxi Zhuang Autonomous Region has important reference value for future HIV/AIDS prevention and control strategies development. The purpose of this study is to analyze the trend of HIV epidemic in Guangxi Zhuang Autonomous Region, and further assess the performance and influence of GACP measures.

Compartment models are one of the most implemented dynamical models in infectious disease [12], where populations are grouped into different compartments according to the infective status, such as susceptible, infectious and recovered. Compartmental models are also widely used in the HIV/AIDS epidemic progression [13], prevention [14–16], intervention [16, 17], control [18–21] and prediction [22] and has great application value in the design of public health policies [23]. Rana et al. [21] construct and analyze a deterministic Susceptible-Infected model for assessing the effect of antiretroviral treatment. Their stability analysis with the basic reproduction number shows that the number of AIDS patients will decrease by receiving proper early antiretroviral treatment. Marsudi et al. [20] formulate an HIV/AIDS transmission compartment model to study the optimal control strategies. By the stability and optimal control analysis, the authors show that the simultaneous implementation of preventive and screening strategies is the best cost effective strategy. Mukandavire et al. [16] demonstrate the use of sex-structured HIV/AIDS compartment models in assessing the effectiveness of condom use as a preventative strategy in a heterosexually active population. Nyabadza et al. [14] propose and analyze a HIV/AIDS model incorporating public-health information compaigns. The results demonstrate that an increase in the rate of dissemination of effective public-health information campaigns results in a decrease in the prevalence of the disease. The results of those studies are based mainly on the stability analysis of the dynamical models, which lacks the support of surveillance data under these implementations.

In this paper, based on the surveillance data of HIV/AIDS case reports, testing and treatment data from Guangxi Zhuang Autonomous Region, we explore the effects of the three measures of GACP project by construction and fitting of a Susceptible-Infected-Diagnosed-Treated compartment model. The three measures include prevention (improvements of the coverage of HIV/AIDS education and reinforcement of behavior intervention), HIV testing (promotion of HIV voluntary counseling and testing, and expansion of HIV testing in medical institutions), and enhancement of ART. In the model, the populations are grouped into the susceptible population, the HIV-infected population (HIV-positive), the diagnosed HIV-positive population, the population under antiretroviral treatment and the population who drop out of the treatment. To effectively simulate the spreading and controlling dynamics of the HIV, we set some parameters in the model to be time dependent, such as the prevention rate, the diagnosis rate and the ART enrolment rate. Based on the HIV surveillance data and the compartment model, we establish a mathematical computational framework for estimating the parameters of the model, evaluation of the basic reproduction number and assessment of the HIV/AIDS epidemic in Guangxi Zhuang Autonomous Region. The Markov-Chain Monte-Carlo method and Nonlinear Least Squares method are applied for parameter estimation in the computational framework. Based on the computational framework, the effects of the three measures of GACP project are evaluated.

Although there are many studies that apply mathematical models to investigate the prevention and control of the disease, there is still not much research on the mathematical model of HIV/AIDS transmission dynamics based on the real-world surveillance data. In this paper, the data-driven mathematical model is well applied to predict the AIDS epidemic situation in Guangxi Zhuang Autonomous Region, and moreover, the implementation effect of the prevention (publicity and intervention), testing and ART measures are well evaluated simultaneously based on the surveillance data and the model. The results of the study could provide reference for developing future provincial HIV/AIDS prevention and control strategies for Guangxi Zhuang Autonomous Region and other provinces with similar epidemic situations.

A list of abbreviations and terms used in the paper is given in Table 1.

**Table 1:** A list of the abbreviations used in the paper.

Term	Meaning
AIDS	Acquired Immune Deficiency Syndrome
HIV	Human Immunodeficiency Virus
GACP	Guangxi AIDS Conquering Project
ART	Antiretroviral Treatment
MCMC	Markov-Chain Monte-Carlo

## Materials and methods

### Data

The data used in this paper comes from the National HIV/AIDS Comprehensive Information System of China’s disease prevention and control information system and the data are not publicly available. The population data comes from the published data of Guangxi Statistical Bureau [24]. The data including HIV/AIDS case report, treatment and intervention data, are collected by local health institutions, including county and township CDC, hospitals and clinics, and reported to Guangxi CDC and China CDC through prefectures and municipal health institutions. Those data included in this study are summary statistics, which do not contain any identifiers that could link the data to individual subjects in the local HIV/AIDS surveillance systems. The mathematical modeling framework is approved by the Guangxi Institutional Review Board (GXIRB2015–0008).

### The mathematical model

**Fig 1:**
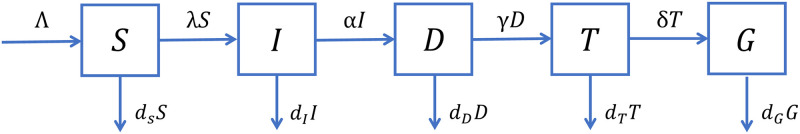
Schematic diagram of the model. Five classes of individuals: Susceptible individuals (*S*), HIV-infected but not yet diagnosed (*I*), diagnosed HIV cases but not yet initiated ART (*D*), initiated ART (*T*), dropped out of ART (*G*). ART: Antiretroviral treatment.

We develop a compartmental model for the HIV transmission dynamics with differential equations. We group the total population into five compartments: The susceptible individuals (*S*), the HIV-infected individuals (HIV-positive) (*I*), the diagnosed HIV-positive individuals (*D*), the individuals under antiretroviral treatment (*T*), and the individuals who drop out of the treatment (*G*). There are three main stages of HIV infection, that is, acute infection, clinical latency, and AIDS. Most HIV infected individuals will eventually progress to AIDS in the absence of treatment [25]. The HIV-infected compartment (*I*) includes all the individuals in the three infection stages. The schematic diagram for our model is shown in Fig 1.

The compartments *S*, *I*, *D*, *T* and *G* are chosen based on the natural history of HIV infection and the available data. Susceptible people (*S*) become infected through contacts with HIV-positive people. HIV-positive people (*I*) are diagnosed through HIV testing. HIV-positive people who are diagnosed (*D*) are then enrolled into the ART program (*T*), some of which drop out of treatment (*G*). The compartments are illustrated in Table 2.

**Table 2:** List of variables.

Notation	Description
*S*	Susceptible individuals
*I*	HIV infected but not yet diagnosed
*D*	Diagnosed HIV-positive individuals who have not yet initiated ART
*T*	Individuals under ART
*G*	Individuals who drop out of ART
*N*	Total population size

We denote the number of susceptible people at time *t* by *S*(*t*), the number of HIV-positive people who are not diagnosed at time *t* by *I*(*t*), the number of diagnosed HIV-positive people who are not under ART at time *t* by *D*(*t*), the number of diagnosed HIV-positive people that are under ART at time *t* by *T*(*t*), and the number of people who drop out of ART at time *t* by *G*(*t*). The time unit used in the model is per year to align with the available data.

Susceptible individuals (*S*) are assumed to be recruited at rate Λ and undergo death at rate *d*_*S*_. Susceptible individuals become infected through contacts with HIV-positive individual (*I*) in an incidence λ(*t*)*S*(*t*), where *N*(*t*) indicates the total population size *N*(*t*) = *S*(*t*) + *I*(*t*) + *D*(*t*) + *T*(*t*) + *G*(*t*). The dynamic of the susceptible individuals (*S*) is given by

$$\frac{dS}{dt}=\Lambda -d_{S}S\left(t\right)-\lambda \left(t\right)S\left(t\right),$$

where the infection incidence λ(*t*) is related to the transmission rate of HIV and the contact rates with infectious compartments, including the infected (*I*), diagnosed (*D*) and treated (*T*) and those drop out of the treatment (*G*). Here we take

$$\lambda \left(t\right)=\frac{c\left(1-\eta \left(t\right)\right)\left(\beta_{I}I\left(t\right)+\beta_{D}D\left(t\right)+\beta_{T}T\left(t\right)+\beta_{G}G\left(t\right)\right)}{N(t)},$$

where *c* denotes the contact rate, and *β*_*I*_, *β*_*D*_, *β*_*T*_ and *β*_*G*_ are transmission coefficients of compartments *I*, *D*, *T* and *G*, respectively. The improvement of the coverage of HIV/AIDS education and the prevention measures could reduce the contact rate, which is indicated by the term 1 − *η*(*t*).

The infected individuals (*I*) either undergo death at rate *d*_*I*_, or are diagnosed through HIV testing at rate *α*(*t*). The dynamics of *I*(*t*) reads

$$\frac{dI}{dt}=\lambda \left(t\right)S\left(t\right)-d_{I}I\left(t\right)-\alpha \left(t\right)I\left(t\right).$$

The promotion of HIV voluntary counseling and testing measures in the GACP project has effect on the HIV testing rates *α*(*t*), so we assume it to be time-dependent.

The diagnosed individuals (*D*) either undergo death at rate *d*_*D*_ or are enrolled into the ART program at rate *γ*(*t*). The dynamics of *D*(*t*) follows

$$\frac{dD}{dt}=\alpha \left(t\right)I\left(t\right)-d_{D}D\left(t\right)-\gamma \left(t\right)D\left(t\right),$$

where *α*(*t*)*I*(*t*) is the annual number of new reports. The enhancement of the antiretroviral treatment would have influence on the treatment enrolment rate *γ*(*t*), which is assumed to be time dependent.

The individuals under antiretroviral treatment (*T*) either undergo death at rate *d*_*T*_ or drop out of treatment at rate *δ*, thus

$$\frac{dT}{dt}=\gamma \left(t\right)D\left(t\right)-d_{T}T\left(t\right)-\delta T\left(t\right),$$

where *γ*(*t*)*D*(*t*) is the number of new treatment enrollments. The drop-outs include those due to treatment failure or loss of follow-up. The drop-out individuals (*G*) undergo death at rate *d*_*G*_, so that

$$\frac{dG}{dt}=\delta T\left(t\right)-d_{G}G\left(t\right).$$

The meanings and values of the parameters are illustrated in Table 3.

**Table 3:** Parameter descriptions, values, 95% CI, and sources for mathematical model.

Notation	Description	Value	95% CI	Source
Λ	Influx of susceptible	720000	-	Database
*d*_*S*_	Death rate of *S*	0.0054	-	Database
*d*_*I*_	Death rate of *I*	0.0630	(0.0676,0.3731)	Estimated
*d*_*D*_	Death rate of *D*	0.1104	(0.0939,0.4107)	Database
*d*_*T*_	Death rate of *T*	0.0167	-	Database
*d*_*G*_	Death rate of *G*	0.0235	-	Database
*δ*	ART dropout rate	0.0566	-	Database
*β* = *cβ*_*I*_	Transmission probability of *I*	0.2602	(0.0411,0.2005)	Estimated
*q*_1_ = *cβ*_*D*_/*β*_*I*_	Ratio of transmission probability	0.75	(0.1837,0.8169)	Estimated
*q*_2_ = *cβ*_*T*_/*β*_*I*_	Ratio of transmission probability	0.075	(0,0.2005)	Estimated
*q*_3_ = *cβ*_*G*_/*β*_*I*_	Ratio of transmission probability	0.1	-	Estimated
*η*(*t*)	Protection rate	0.00420(*t* − 2005) + 0.0052 if 2005 ≤ *t* < 2010 0.00126(*t* − 2005) − 0.0284 if *t* ≥ 2010		Estimated
*α*(*t*)	Diagnosis rate	0.0560(*t* − 2005) + 0.5743 if 2005 ≤ *t* < 2010 0.0665(*t* − 2005) + 0.5343 if *t* ≥ 2010		Estimated
*γ*(*t*)	ART enrolment rate	0.0062(*t* − 2005) + 0.1014 if 2005 ≤ *t* < 2010 0.0225(*t* − 2005) + 0.0362 if *t* ≥ 2010		Database & Estimated
*S*(0)	Initial number of *S*	49232869	-	Database
*I*(0)	Initial number of *D*	14000	(2436,17538)	Estimated
*D*(0)	Initial number of *I*	16696	-	Database
*T*(0)	Initial number of *T*	435	-	Database
*G*(0)	Initial number of *G*	18	-	Database
*R* ^2^	Coefficient of determination	0.90	-	-

With the implementation of the Four Frees and One Care program in 2003, the Chinese government had rapidly scaled up HIV testing and ART, which was reflected by an increase in the number of HIV tested people and in the number of treatment centers. The surveillance data for Guangxi Zhuang Autonomous Region is available from the year 2005. The 5-year Guangxi AIDS Conquering Project (GACP) was launched from 2010. To correctly adjust for the increase in new HIV testing and ART, we use a time-dependent prevention rate *η*(*t*), diagnosis rate *α*(*t*), ART enrolment rate *γ*(*t*) and model them by continuous piecewise linear functions. We assume that

$$\begin{array}{c} \eta \left(t\right)=\{\begin{array}{cc} \eta_{11}\left(t-2005\right)+\eta_{12} & \text{if}\;2005\leq t<2010,\\ \eta_{21}\left(t-2005\right)+\eta_{22} & \text{if}\;t\geq 2010, \end{array} \end{array}$$

$$\begin{array}{c} \alpha \left(t\right)=\{\begin{array}{cc} \alpha_{11}\left(t-2005\right)+\alpha_{12} & \text{if}\;2005\leq t<2010,\\ \alpha_{21}\left(t-2005\right)+\alpha_{22} & \text{if}\;t\geq 2010. \end{array} \end{array}$$

$$\begin{array}{c} \gamma \left(t\right)=\{\begin{array}{cc} \gamma_{11}\left(t-2005\right)+\gamma_{12} & \text{if}\;2005\leq t<2010,\\ \gamma_{21}\left(t-2005\right)+\gamma_{22} & \text{if}\;t\geq 2010, \end{array} \end{array}$$

where *γ*(*t*) will be fitted with ART enrollment surveillance data.

### The basic reproduction number

Our model was used to calculate the reproduction number *R*_*C*_, which is the actual average number of secondary cases per primary case caused observed in a population for an infectious disease in the presence of control measures. The reproduction number includes the effect of intervention measures, which varies as the epidemic progresses with time. Since our model parameters are time dependent, the reproduction number is suitable indicator of the strength of the transmission dynamics.

In our model, effects of the national programs (Four Frees and One Care) after 2003 and GACP in Guangxi Zhuang Autonomous Region after 2010 are incorporated into the time dependent prevention rate *η*(*t*), diagnosis rate *α*(*t*) and ART enrolment rate *γ*(*t*). The impact of the programs during the time period 2005–2020 is measured by the time-varying reproduction number *R*_*C*_

$$\begin{array}{c} R_{C}\left(t\right)=\left(1-\eta \left(t\right)\right)\frac{\beta }{d_{I}+\alpha \left(t\right)}(1+\frac{q_{1}\alpha \left(t\right)}{d_{D}+\gamma \left(t\right)}+\frac{q_{2}\alpha \left(t\right)\gamma \left(t\right)}{\left(d_{D}+\gamma \left(t\right)\right)\left(d_{T}+\delta \right)}\\ +\frac{q_{3}\alpha \left(t\right)\gamma \left(t\right)\delta }{\left(d_{D}+\gamma \left(t\right)\right)\left(d_{T}+\delta \right)d_{G}}), \end{array}$$

where *β* = *cβ*_*I*_, *q*_1_ = *cβ*_*D*_/*β*_*I*_, *q*_2_ = *cβ*_*T*_/*β*_*I*_ and *q*_3_ = *cβ*_*G*_/*β*_*I*_. The mathematical derivation of the basic reproduction number and the interpretation are given in Appendix B.

### Framework

**Fig 2:**
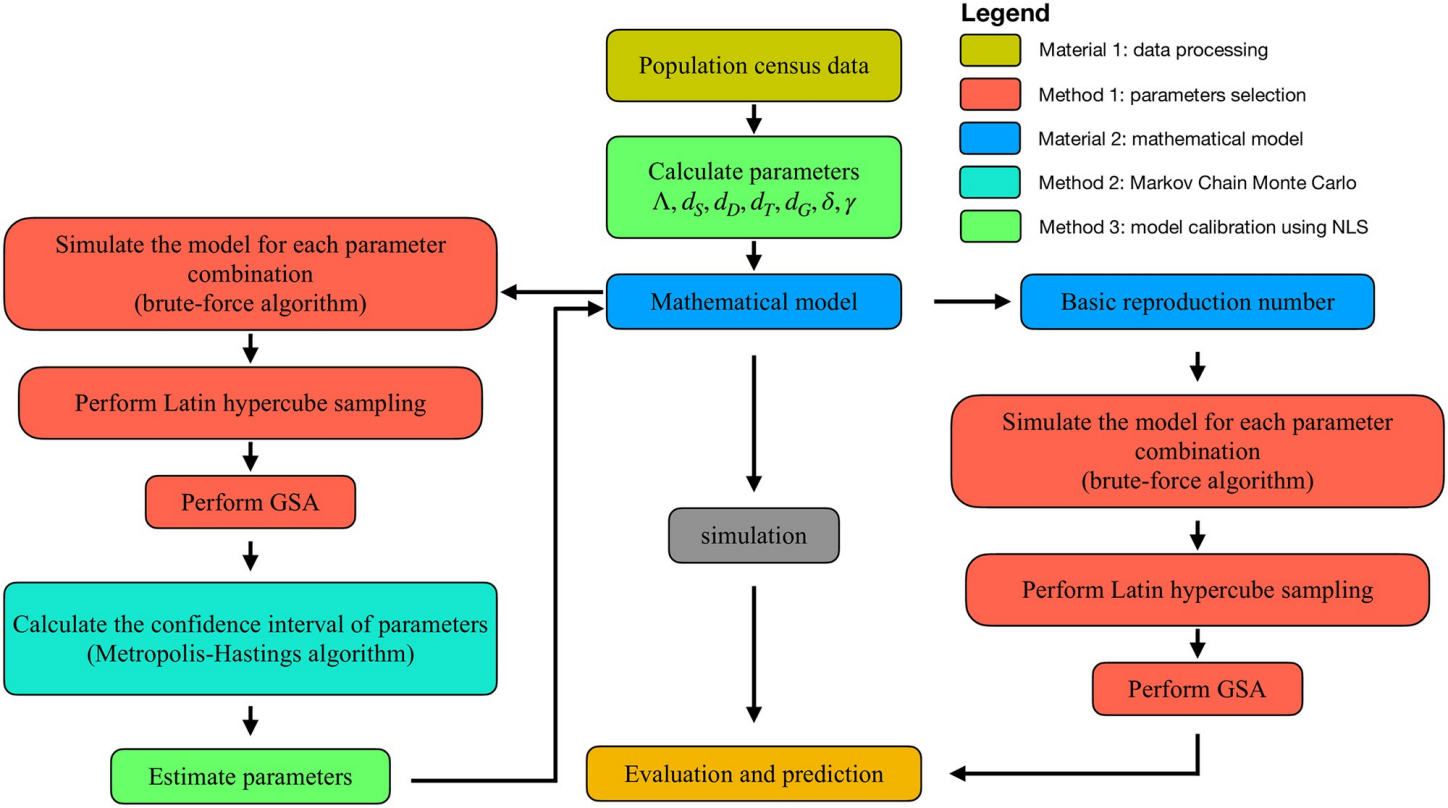
Computational framework. In the first step, we apply nonlinear least square method to estimate parameters (Λ, *d*_*S*_, *d*_*D*_, *d*_*T*_, *d*_*G*_, *δ*, *γ*(*t*)) by surveillance data. Next, with the surveillance data, the probability density function (PDF) of parameters (*d*_*I*_, *d*_*D*_, *I*(2005), *β*, *q*_1_, *q*_2_) are estimated by using Markov-Chain Monte-Carlo (MCMC) method. Then the model parameters are estimated by nonlinear least square method. In the second step, we calculate the reproduction number. In the last step, we try to assess the HIV/AIDS epidemic in Guangxi Zhuang Autonomous Region by calibrating mathematical model and the reproduction number. GSA: Global sensitivity analysis.

In this paper, we construct a computational framework [26] (in Fig 2) to analyze the surveillance data, calibrate the mathematical model, and then evaluate the effect of GACP and put forward suggestions for the prevention and control of HIV/AIDS epidemic in Guangxi Zhuang Autonomous Region.

Firstly, we use the surveillance data to estimate the death rate of the *D*, *T* and *G* population, the dropout rate *δ* and the ART enrolment rate *γ*(*t*) directly. Secondly, we establish differential equations model to describe the dynamic behavior of different groups. Then we calibrate the model parameters by MCMC and nonlinear least square methods. Finally, we try to assess the HIV/AIDS epidemic in Guangxi Zhuang Autonomous Region.

In addition, the reproduction number is obtained by the mathematical model. By analyzing the influence of different prevention and control measures on the reproduction number, we obtain valuable suggestions for the prevention and control of HIV/AIDS epidemic in Guangxi Zhuang Autonomous Region.

### Statistical analysis

#### Parameter estimation using surveillance data

Demographic parameters Λ and *d*_*N*_ are estimated by fitting the equation of the total population

$$\frac{dN}{dt}=\Lambda -d_{S}N,$$

to the population data of Guangxi Zhuang Autonomous Region from 2005 to 2020. We obtain that Λ = 720000 and *d*_*S*_ = 0.0054. The result is shown in Fig 4A.

Values of the parameters *d*_*D*_, *d*_*T*_, *d*_*G*_, *δ*, and *γ*(*t*) are estimated directly from the surveillance data. From the data of total survivals of HIV-positive patients and the deaths, and the total treated and death, we estimate the fatalities of *d*_*D*_ and *d*_*T*_ to be *d*_*D*_ = 0.1134, and *d*_*T*_ = 0.0308. Based on the data of new treatments and drop-outs per year, we estimate the dropout rate to be *δ* = 0.0533, and the ART enrolment rate *γ*(*t*) to be

$$\begin{array}{c} \gamma \left(t\right)=\{\begin{array}{cc} 0.0233(t-2005)+0.0367 & \text{if}\;2005\leq t<2010,\\ 0.0580(t-2005)-0.1295 & \text{if}\;t\geq 2010. \end{array} \end{array}$$ (4)

**Fig 3:**
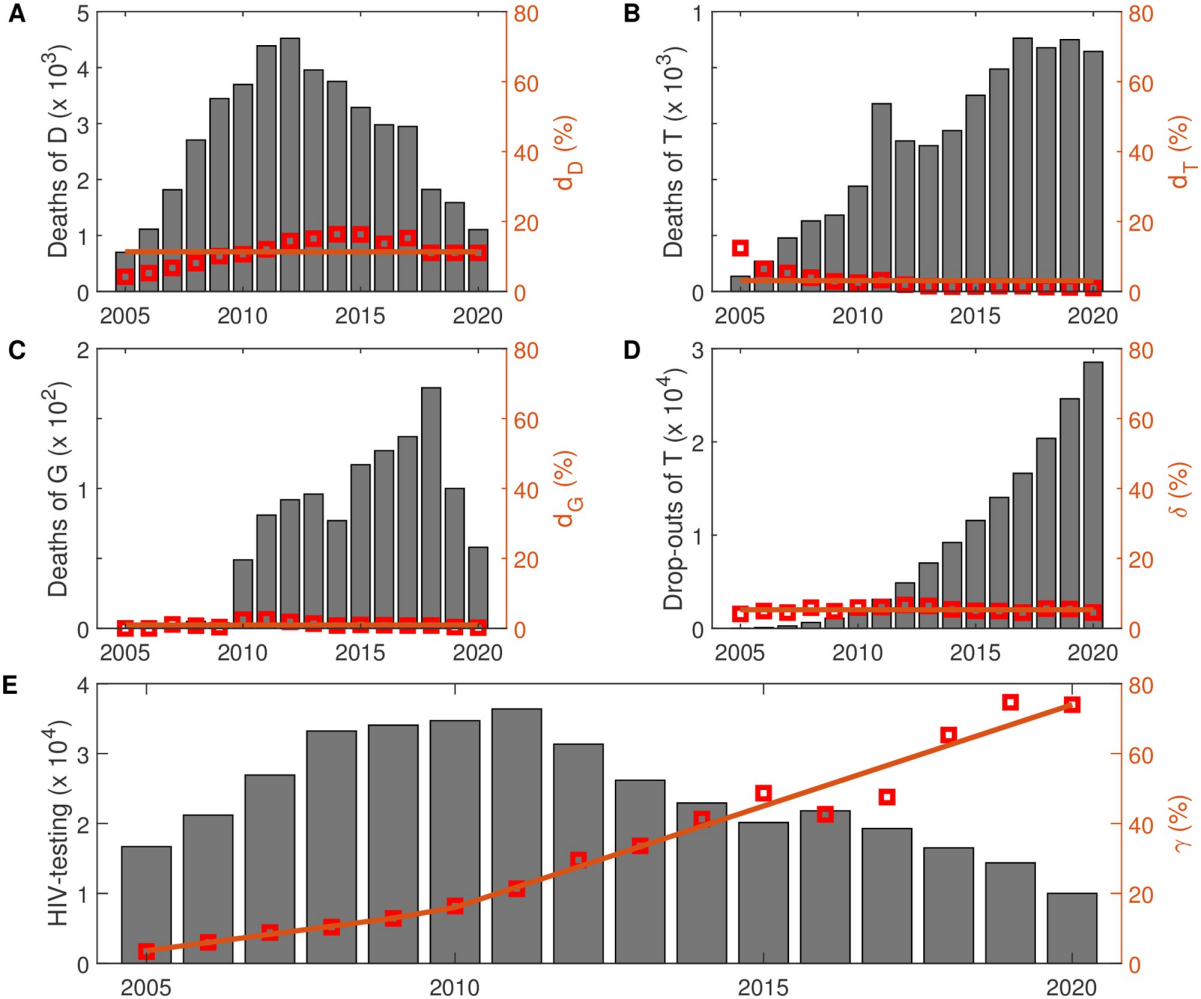
Surveillance data and parameter estimation. The histogram shows the annual deaths of HIV-positive individuals (A), the annual deaths of individuals under ART (B), the annual deaths of individuals who drop out of ART (C), the annual drop-outs of ART (D) and the annual HIV-testing individuals (E). The parameters *d*_*D*_, *d*_*T*_, *d*_*G*_, *δ* and *γ*(*t*) are estimated from the surveillance data of Guangxi Zhuang Autonomous Region from 2005 to 2020 directly (in orange), where *d*_*D*_ = 0.1134, *d*_*T*_ = 0.0308, *d*_*G*_ = 0.0235, *δ* = 0.0098, *γ*(*t*) = 0.0233(*t* − 2005) + 0.0367 before 2010 and *γ*(*t*) = 0.0580(*t* − 2005) − 0.1295 after 2010. The red point is the surveillance data, and the orange line is the estimation result. ART: Antiretroviral treatment.

Time *t* is in unit of year, and the results are shown in Fig 3.

#### Model fitting

The new infection per year is given by

$$\phi \left(t\right)=(1-\eta (t))c\frac{\beta_{I}I+\beta_{D}D+\beta_{T}T+\beta_{G}G}{N}S,$$

that is

$$\phi \left(t\right)=(1-\eta (t))\beta \frac{I+q_{1}D+q_{2}T+q_{3}G}{N}S,$$

where *β* = *cβ*_*I*_, *q*_1_ = *cβ*_*D*_/*β*_*I*_, and *q*_2_ = *cβ*_*T*_/*β*_*I*_.

The nonlinear least squares method is applied to find the point estimates for mathematical model parameters and the initial value for compartment *I* at the end of 2005, which minimize the summation of squared error between model output and the available surveillance data. The estimated values are shown in Table 3.

Before using the nonlinear least squares method, we use the MCMC method [22] to obtain the 95% confidence interval of some parameters. The MCMC method provides the iterative starting point and 95% confidence interval basis for the nonlinear least square method, so as to prevent the nonlinear least square method from falling into the local optimal solution in a large interval and unable to find the optimal solution in a small interval, which can effectively fill the gap between statistical data and the nonlinear least square method. The details of the MCMC method are provided in the Appendix A.

#### Fitting effect

The goodness of the fitting is accounted by coefficient of determination [27]

$$R^{2}=1-\frac{SS_{res}}{SS_{tot}},$$

where *SS*_*res*_ represents the error between the statistical data **y** and the best fit data $\hat{y}$, which is the sum of squares of residuals, also called the residual sum of squares, that is

$$SS_{res}=\sum_{i}{\left(y_{i}-{\hat{y}}_{i}\right)}^{2}$$

and *SS*_*tot*_ indicates the error between the statistical data and the average of the statistical data $\bar{y}$, which is proportional to the variance of the data, that is

$$SS_{tot}=\sum_{i}{\left(y_{i}-\bar{y}\right)}^{2}.$$

**Fig 4:**
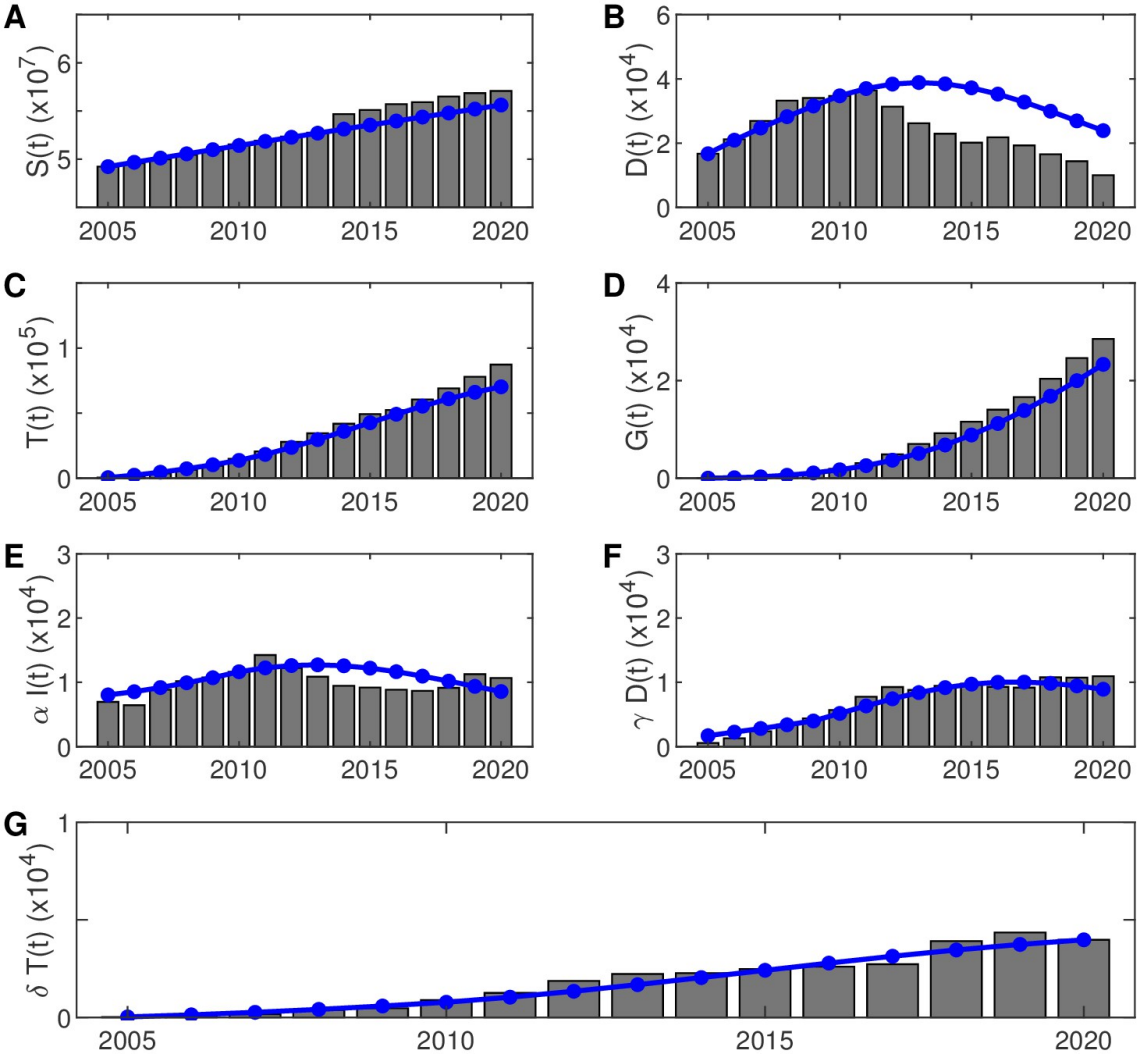
Comparison between model output and surveillance data. Fitting of simulation data (in blue) to statistical data (histogram) from 2005 to 2020. The histogram shows the annual susceptible individuals (A), the annual diagnosis individuals (B), the annual individuals under ART (C), the annual drop-outs of ART (D), the annual new diagnosis individuals (E), the annual new individuals under ART (F), the annual new drop-outs of ART (G). The models were well fitted between the simulated data and the surveillance data (*R*^2^ = 0.90). ART: Antiretroviral treatment.

The models are well fitted between the simulated data and the surveillance data (*R*^2^ = 0.90), and the fitting effects are shown in Fig 4. Here, the simulation data of individuals in the diagnosed compartment is not well fitted with the surveillance data after the implementation of the GACP (see Fig 4B). This may partly due to the data collection and calculation of individuals in the diagnosed compartment, while the annual diagnosed data and annual treatment enrolment data are more reliable which are well fitted as shown in Fig 4E and 4F, respectively.

## Results

### Evaluation of the effects of GACP on the epidemic in Guangxi Zhuang Autonomous Region

Under the implementation of GACP and the National Program (Four Free and One Care), the Guangxi government has strengthened HIV testing, prevention and treatment; the numbers of HIV testing and treatment have greatly increased in Guangxi Zhuang Autonomous Region, and furthermore, the publicity and education were strengthened, as well as the promotion of condoms.

Preventive measures include warning publicity and education, actively promoting various publicity activities, carrying out many publicity lectures, and strengthening the publicity of floating population, in addition, interventions including needle-syringe exchange, Methadone maintenance treatment, peer education, condom promotion are provided to injecting drug users (IDUs); condom promotion and peer education are provided to sex workers and men who have sex with men (MSM). HIV testing measures include voluntary counseling and testing towards high risk groups like IDUs, sex workers and their clients, MSM and spouses of HIV cases, and moreover, the range of HIV testing in medical institutions is expended, such as the addition of pre-operation testing, pre-marital testing, pregnancy and childbirth testing and blood donation testing. Antiviral therapy measures include improving the quality of medical services, free ART, early initiation of ART regardless of their CD4+ counts level.

**Fig 5:**
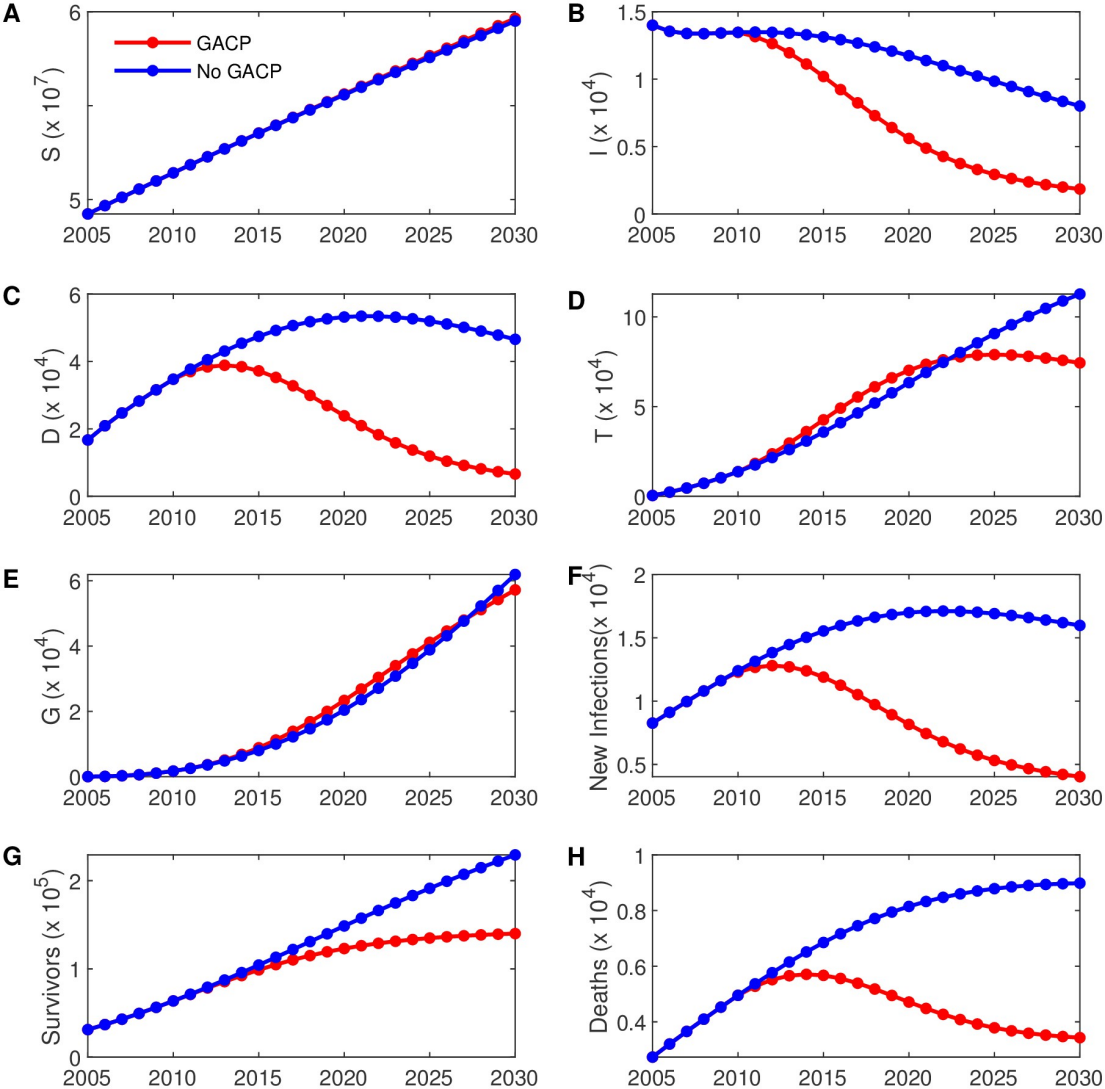
Comparison of the effects of implementing GACP and not implementing GACP. (A)—(H) The growth of (S, I, D, T, G, new HIV/AIDS infections, AIDS survivors, AIDS deaths) without the GACP project (in blue) and with GACP project (in red) from 2005 to 2030.

We utilize the calibrated model to assess the implementation effect of GACP; the results are shown in Fig 5 and Table 4. We can observe that GACP effectively reduced the number of HIV infected people and the number of HIV related deaths. In contrast, the implementation of GACP has little effect on the susceptible people (*S*).

**Table 4:** Estimated data about new infections, survivals and deaths.

	The National Program			The GACP program			Reduction		
	Infection	Survival	Death	Infection	Survival	Death	Infection	Survival	Death
2011	13138	71257	5366	12665	71021	5278	3.5%	0.3%	1.6%
2012	13830	79178	5766	12803	78371	5515	7.4%	1.0%	4.3%
2013	14464	87370	6149	12706	85550	5658	12.1%	2.1%	8.0%
2014	15035	95793	6512	12392	92425	5707	17.5%	3.5%	12.4%
2015	15539	104402	6852	11893	98886	5670	23.5%	5.3%	17.2%
2016	15972	113152	7166	11251	104849	5558	29.6%	7.3%	22.4%
2017	16333	121998	7454	10512	110259	5388	35.6%	9.6%	27.8%
2018	16622	130895	7714	9724	115093	5179	41.5%	12.1%	32.9%
2019	16841	139801	7945	8926	119353	4948	47.0%	14.6%	37.7%
2020	16993	148675	8148	8156	123062	4710	52.0%	17.2%	42.2%

Under the implementation of GACP, the number of HIV-positive people (*I*) decreased significantly. The number of diagnosed people (*D*) reached its peak in 2012 with GACP, while the number of diagnosed people (*D*) is expected to reach its peak in 2023 without GACP. The number of new HIV infections peaked in 2012 with GACP, while it peaked in 2020 without GACP. Similarly, with GACP, the number of HIV/AIDS survivors will peak in 2030, while without GACP, the number of HIV/AIDS survivors will peak in 2050. With GACP, the number of AIDS deaths peaked in 2014, while without GACP, the number of AIDS deaths will peak in 2030.

At the initial stage of GACP implementation, the number of people initiated ART (*T*) is briefly higher than that without GACP (from 2010 to 2022). This is due to the implementation of GACP, that is, the potential HIV/AIDS patients are treated; with the advancement of time, the number of HIV/AIDS patients receiving treatment effectively increased. Similarly, in the process of implementing GACP, the number of people who drop out of ART (*G*) is temporarily higher than the number of *G* without GACP (from 2010 to 2022). This maybe due to the increase in the number of AIDS patients receiving treatment, and more AIDS people giving up treatment for various reasons, such as economic, suicide [28]. With the continuous implementation of GACP, the number of patients giving up ART has been effectively controlled.

According to our model estimates, through the implementation of GACP, Guangxi Zhuang Autonomous Region had reduced 52% newly reported HIV infections, 17.2% HIV/AIDS survivors and 42.2% deaths in 2020, as shown in Table 4. At the same time, we find that from 2010 to 2020, the amount of the above reduced percentage are increasing every year.

### Sensitive analysis for *R*_*C*_ and alternative intervention scenarios

**Fig 6:**
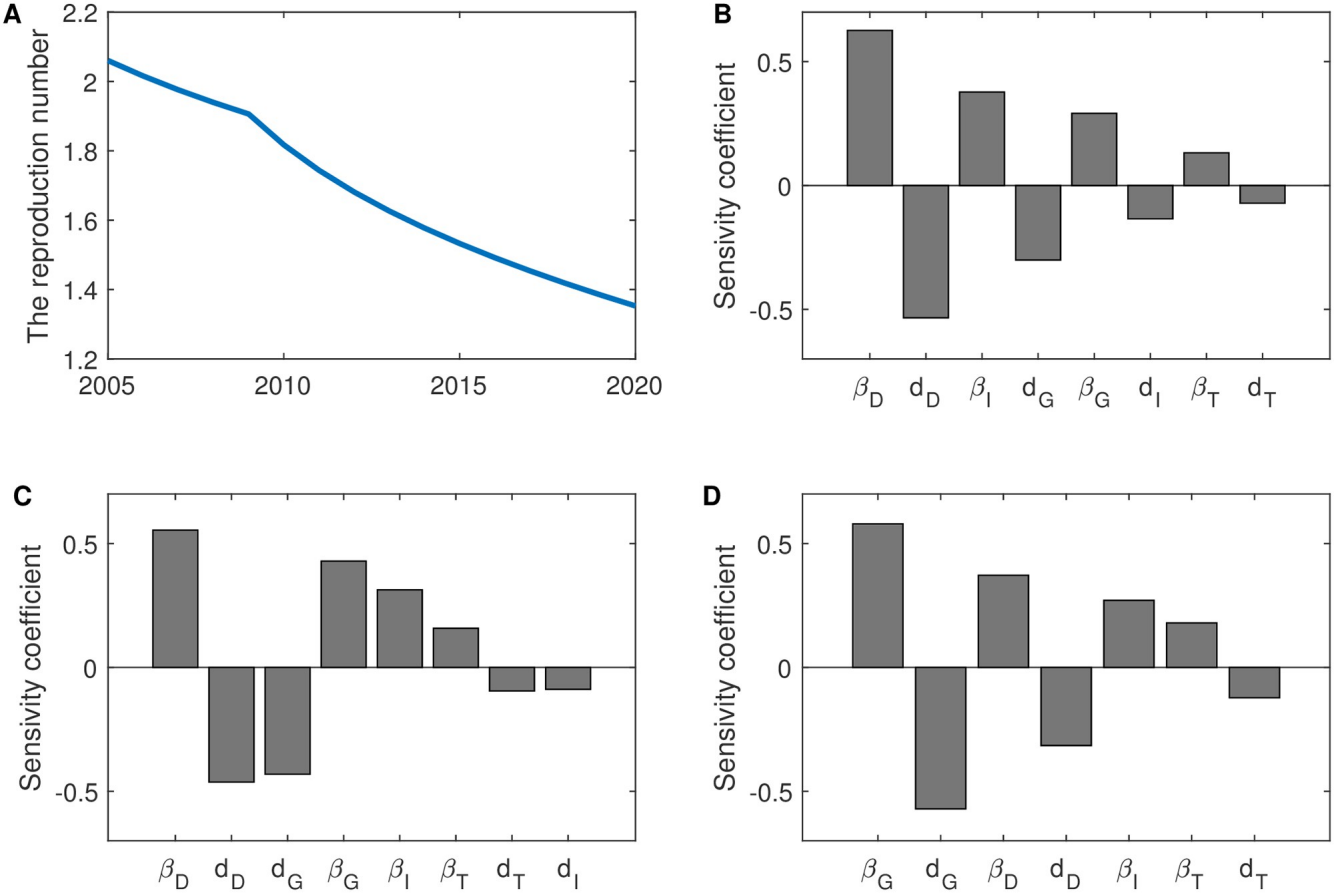
Estimated annual value of the reproduction number *R*_*C*_ and its sensitivity analysis. (A) The estimated value of *R*_*C*_ from 2005 to 2020. Sensitivity analysis results of *R*_*C*_ in 2005 (B), 2010 (C) and 2015 (D). Latin hypercube sampling was used to analyze the sensitivity of 10,000 samples. All parameter values are the same as in Table 5.

We use Latin Hypercube Sampling method to analyze the sensitivity of parameter on the reproduction number; the results of the sensitive analysis for year 2005, 2010 and 2015 are shown in Fig 6. The ranges of parameter values used for sensitive analysis are shown in Table 5.

**Table 5:** Ranges of model parameter values for sensitive analysis.

Parameter	Description	Value	Range
*d*_*I*_	Death rate of *I*	0.0630	(0.0599,0.0662)
*d*_*D*_	Death rate of *D*	0.1104	(0.1049,0.1159)
*d*_*T*_	Death rate of *T*	0.0167	(0.0158,0.0175)
*d*_*G*_	Death rate of *G*	0.0235	(0.0223,0.0247)
*β* = *cβ*_*I*_	Transmission probability of *I*	0.2602	(0.2471,0.2732)
*q*_1_ = *cβ*_*D*_/*β*_*I*_	Ratio of transmission probability	0.75	(0.5,1)
*q*_2_ = *cβ*_*T*_/*β*_*I*_	Ratio of transmission probability	0.075	(0.5,1)
*q*_3_ = *cβ*_*G*_/*β*_*I*_	Ratio of transmission probability	0.1	(0.05,0.15)

In 2005, the most sensitive parameters for *R*_*C*_ are the transmission coefficient *β*_*D*_ and death rate *d*_*D*_ of the diagnosed population (compartment *D*). As HIV testing and ART coverage increases, more HIV-positive people are diagnosed and treated, in the case of limited medical resources, which also means that more patients drop out treatment (for economic reasons, suicide reasons [28] or other reasons). Therefore, in 2010, the sensitivity of transmission coefficient *β*_*D*_ and death rate *d*_*D*_ to *R*_*C*_ gradually decreased in the diagnosed population (compartment *D*), while the sensitivity of transmission coefficient *β*_*G*_ and death rate *d*_*G*_ gradually increased in the population dropped out of ART (compartment *G*). In 2015, with the increase in the number of drop-outs, the transmission coefficient *β*_*G*_ and death rate *d*_*G*_ of the population dropped out of ART (compartment *G*) became highly sensitive to *R*_*C*_.

**Fig 7:**
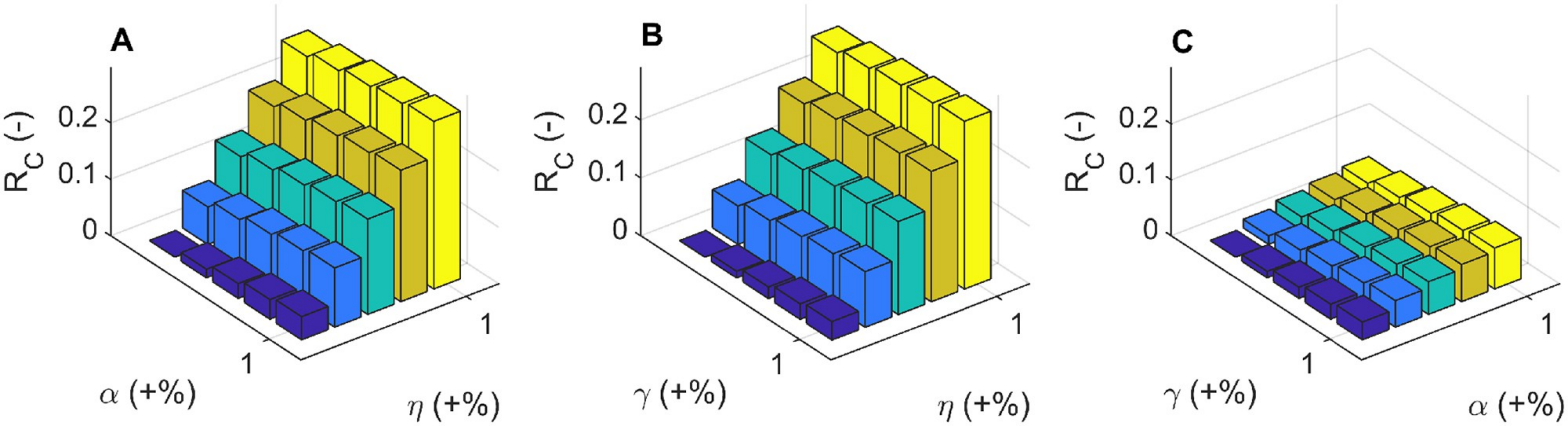
The reduction of the reproduction number (*R*_*C*_(−)). The reduction of the reproduction number (*R*_*C*_(−)) under different combinations of the improvement of diagnosis rate (*α*(+ %), the improvement of protection rate (*η*(+ %)), and the improvement of ART enrolment rate (*γ*(+ %)). (A) *R*_*C*_(−) under the combination of *α*(+ %) and *γ*(+ %). (B) *R*_*C*_(−) under the combination of *α*(+ %) and *η*(+ %). (C) *R*_*C*_(−) under the combination of *γ*(+ %) and *α*(+ %). ART: Antiretroviral treatment.

In order to further investigate the impact of prevention, diagnosis and treatment measures on HIV/AIDS epidemic, we compared the effects of the three different measures on the reduction of the basic reproduction number *R*_*C*_(−). In contrast to the improvement of diagnosis rate *α*(+ %) and ART enrolment rate *γ*(+ %), the improvement of the protection rate (education rate) *η*(*t*) has a great influence on the reduction of the reproduction number *R*_*C*_, as shown in Fig 7.

We further take the non-implementation of GACP as the control sample, to compare different implementation scenarios applying the calibrated model. Compared with the control sample, we construct seven hypothetical scenarios, as shown in Table 6. Scenario 7, for example, includes antiretroviral treatment under the national program, prevention and HIV testing under the GACP program, and so on in other scenarios.

**Table 6:** List of different implementation scenarios.

	The GACP Program			Estimated *R*_*C*_ in 2020	Reduction (%)	New infection in 2020	Reduction (%)
	Prevention	Diagnosis	Treatment				
Scenario 1	-	-	-	1.6510	-	16994	-
Scenario 2	✔	-	-	1.4872	9.9	13320	21.6
Scenario 3	-	✔	-	1.6413	0.6	16600	2.3
Scenario 4	-	-	✔	1.5117	8.4	10665	37.2
Scenario 5	✔	✔	-	1.4786	10.4	13029	23.3
Scenario 6	✔	-	✔	1.3618	17.5	8387	50.6
Scenario 7	-	✔	✔	1.5016	9.0	10352	39.1
Scenario 8	✔	✔	✔	1.3527	18.1	8156	52.0
Remark	Take Scenario 1 as the control case.						

**Fig 8:**
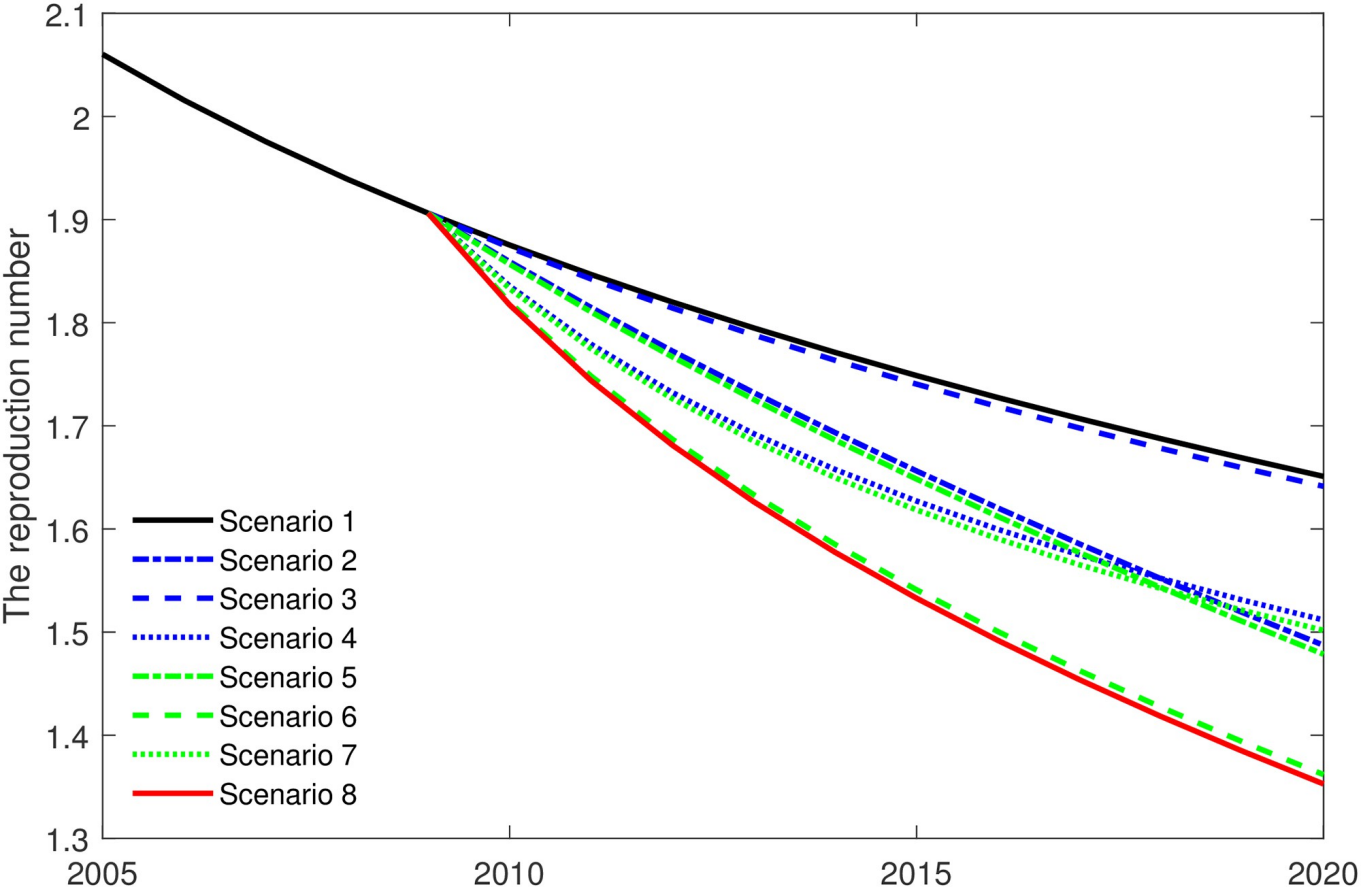
Estimation of the impact of various implementation scenarios on the value of the reproduction number *R*_*C*_. The maximum reduction (to 1.3527) and the minimum reduction (to 1.6413) of *R*_*C*_ are achieved by Scenario 8 and scenario 3, respectively. The scenarios are illustrated in Table 6.

The simulation results are shown in Fig 8 and Table 6. The maximum reduction (to 1.3527) and the minimum reduction (to 1.6413) of *R*_*C*_ are achieved by Scenario 8 and Scenario 3, respectively. In addition, we find that that there is little difference between Scenario 1 (to 1.6510) and Scenario 3 (to 1.6413) for reduction of the reproduction number. Similarly, Scenario 2 (to 1.4872) and Scenario 5 (to 1.4786), Scenario 4 (to 1.5117) and Scenario 7 (to 1.5016), Scenario 6 (to 1.3618) and Scenario 8 (to 1.3527) have similar effects. That is to say, diagnosis *α* has some effect on the reproduction number *R*_*C*_ in the former period, but later on has little influence on the reproduction number *R*_*C*_ compared with prevention *η* and treatment *γ*.

In addition, we simulate the number of new HIV infections (in 2020) with different scenarios. We observe that Scenario 4 (37.2%) has a better effect on reducing the number of new HIV infections than Scenario 2 (21.6%) and Scenario 3 (2.3%). It is worth mentioning that although ART could effectively reduce the number of new infections, it is on the premise of large-scale HIV testing. Without large-scale HIV testing, ART cannot be accurately administered to the target population.

## Discussion

The current studies of AIDS epidemics lack research on the evaluation of large-scale AIDS control projects based on surveillance data. Our study is the first to model and evaluate the implementation effect of the GACP project based on surveillance data in Guangxi Zhuang Autonomous Region, China. The GACP project is an effective attempt to prevent and control AIDS by the local government in China. Our research provides abundant surveillance data, prediction data and model support for the AIDS epidemic in Guangxi Zhuang Autonomous Region. Furthermore, it provides an important reference for future AIDS epidemic control and public health policies in developing regions.

In order to estimate the epidemic trend of HIV/AIDS in Guangxi Zhuang Autonomous Region, China, we establish a mathematical model assuming homogeneous population mixing in different cities in this area based on the surveillance data. In particular, in order to access the impact of HIV/AIDS transmission in different populations, the mathematical model is a Susceptible-Infected-Diagnosed-Treated population compartments model. Based on this model, we put forward a mathematical framework for evaluation, including analysis of surveillance data, calibration of mathematical model, calculation of the reproduction number, prediction of the HIV/AIDS epidemic and evaluation of the effectiveness of the GACP program.

We observe that the GACP program effectively reduced the number of HIV infections and the number of HIV related deaths. In contrast, the implementation of GACP has little effect on the susceptible people, which is reasonable, since the implementation of GACP will not affect the natural growth of the population. The basic reproduction number *R*_*C*_ is derived from the mathematical model and estimated based on HIV/AIDS surveillance data in Guangxi Zhuang Autonomous Region. The sensitivity analysis (as shown in Fig 6) show that the transmission coefficients *β*_*G*_ and death rate of people who drop out of ART (compartment *G*) have a great influence on *R*_*C*_ over time. Further numerical simulations (as shown in Fig 7) show that measure of the HIV-testing as a single-strategy would have a limited impact on the reduction of the basic regeneration number *R*_*C*_, that is, increasing in HIV testing rates *α*(*t*) alone while maintaining the prevention rate *η*(*t*) and treatment rates *γ*(*t*), would have only a slight impact on the number of HIV/AIDS cases in the near future. This result further implies that how to promote HIV/AIDS publicity and education and improve the ART enrollment rate has become an important issue in HIV/AIDS prevention and treatment. Therefore, it is important for the Guangxi government to retain the current free antiretroviral treatment, and try to allocate treatment resources to allow HIV/AIDS patients to receive continuous antiretroviral treatment.

In order to estimate the parameters more accurately, we apply the classical machine learning method, namely the MCMC method [22, 29]. Admittedly, our results have some limitations. First of all, the estimation results of this study are based on surveillance data. We ignore the specific transmission mode of HIV in the model. In fact, after 2005, the main mode of HIV transmission in Guangxi Zhuang Autonomous Region has changed greatly. Before 2006, the mode of HIV transmission in Guangxi was mainly caused by injecting drugs, while since 2006, heterosexual sex has become the dominant mode of HIV transmission, followed by drug injection [30]. The estimation results using a simplified model may underestimate the transmission of HIV. Secondly, our model does not consider the heterogeneity of the HIV-positive population. For example, the estimated values of transmission rates *β*_*I*_, *β*_*D*_, *β*_*T*_, *β*_*G*_ are the average values; the age structure [31] and sex structure [16] of the population are not considered. For example, older heterosexual adults seem to have a higher risk of HIV infection [30, 32]. In view of the significant spatial variability of HIV infection, the reproduction number under heterogeneous mixing may be greater than that under homogeneous mixing. The model needs further development and refinement to take into account the heterogeneity within different groups in order to make a more accurate estimate of the HIV/AIDS epidemic. In addition, as the expansion of antiretroviral treatment is expected to improve the survival rate of HIV/AIDS patients, the death rate of patients receiving antiretroviral treatment is expected to decrease over time (as shown in Fig 3B). However, we adopt a constant death rate in our model, which may lead to a slight overestimation of *R*_*C*_. Finally, it should be noted that our Susceptible-Infected-Diagnosed-Treated population compartments model is idealized and does not consider the spatial factor, that is, the mobility of AIDS patients between provinces; Guangxi Zhuang Autonomous Region is located at the border between the two countries, so our model does not consider the transmission input from other countries (such as the Golden Triangle region). Therefore, the model could be improved to a patch model between different regions.

However, our results strongly suggest that the implementation of GACP will lead to an overall decline in HIV infection. Therefore, enhanced interventions and local government-based HIV/AIDS support programs should be widely implemented in areas with severe epidemics to effectively reduce HIV spreading in the Chinese mainland.

## Appendix A. Markov Chain Monte Carlo method

Markov chain Monte Carlo method [22, 29] was used to obtain 95% confidence intervals for model parameters *β*_*I*_, *d*_*I*_, *d*_*D*_, *q*_1_, *q*_2_, *I*_0_. We take an example of the process of obtaining the 95% confidence interval of parameter *β*_*I*_. It can be obtained for other parameters in the same way.

### Sensitive analysis of SSE

Let *SSE*(*β*_*I*_) is the sum of squared errors between statistical data **y** and model output $\hat{y}\left(\beta_{I}\right)$, that is,

$$SSE\left(\beta_{I}\right)=\sum_{i=1}^{N}\left(y_{i}-{\hat{y}}_{i}\left(\beta_{I}\right)\right)$$

where *N* is the number of data points.

We performed sensitivity analysis with respect to *SSE* for the parameters *β*_*I*_, *d*_*I*_, *d*_*D*_, *q*_1_, *q*_2_, *I*_0_. Following the method of Latin Hypercube Sampling, we generated 100,000 samples to calculate the Pearson correlation coefficient between each parameter combinations and *SSE*.

**Fig 9:**
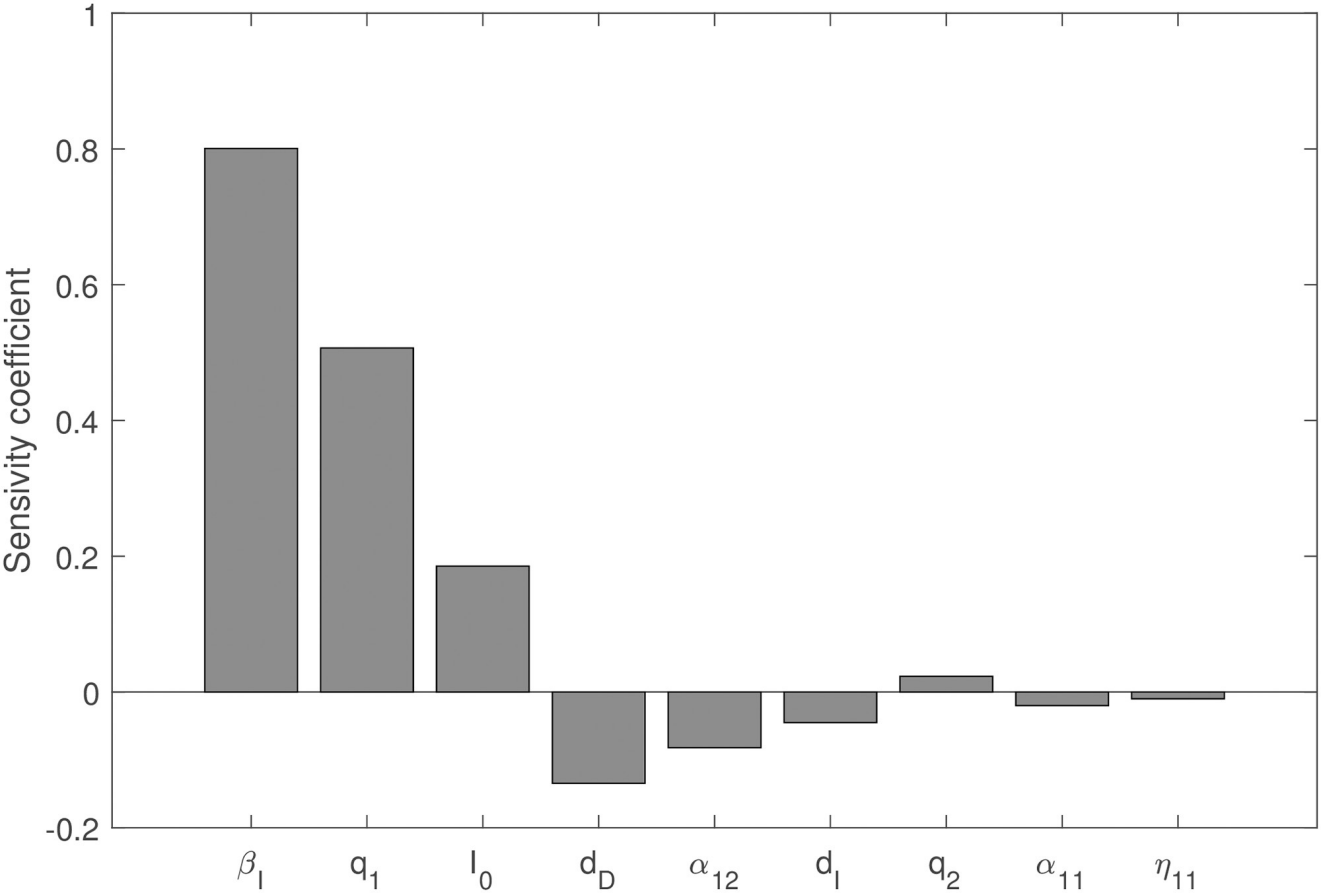
The sensitivity analysis of *SSE*. Latin Hypercube Sampling method is used to analyze the sensitivity of 100,000 samples. All parameter values are the same as in Table 3.

The results are shown in Fig 9.

We see from Fig 9 that *β*_*I*_, *q*_1_, *I*_0_, *q*_2_ are all positively correlated with *SSE*. All other parameters are negatively correlated to *SSE*.

### The posterior distribution of parameter *β*_*I*_ from data y

Assume that the errors introduced in statistical data are normally distributed, that is,

$$y=\hat{y}\left(\beta_{I}\right)+\epsilon ,$$

where $\hat{y}\left(\beta_{I}\right)$ is the model output given the parameter *β*_*I*_, **ϵ** is a random variable with multivariate normal distribution, that is

ϵ∼N(0,Σ),

where Σ = *diag*(*σ*^2^, *σ*^2^, …, *σ*^2^) and *σ*^2^ is a random variable with inverse Gamma distribution.

Based on the above assumptions, the likelihood function is calculated as follows

$$p\left(y|\beta_{I}\right)=\int p\left(y|\beta_{I},\sigma^{2}\right)p\left(\sigma^{2}\right)d\sigma^{2},$$

where $p\left(\sigma^{2}\right)=\frac{\beta_{\alpha }}{\Gamma (\alpha )}\sigma^{-2\alpha -2}e^{\frac{\beta }{\sigma^{2}}}$, since *σ*^2^ ∼ *Inv*Γ(*α*, *β*).

According to the properties of the probability density function of the multivariate normal distribution,

$$p\left(y|\beta_{I},\sigma^{2}\right)=p\left(y_{i}|\beta_{I},\sigma^{2}\right)p\left(y_{i}|\beta_{I},\sigma^{2}\right)\ldots p\left(y_{N}|\beta_{I},\sigma^{2}\right)={\left(2\pi \sigma^{2}\right)}^{-\frac{N}{2}}e^{-\frac{\Sigma_{i=1}^{N}{\left(y_{i}-{\hat{y}}_{i}\left(\beta_{I}\right)\right)}^{2}}{2\sigma^{2}}},$$

we can obtain that

$$p\left(y|\beta_{I}\right)=\int {\left(\frac{1}{2\pi \sigma^{2}}\right)}^{\frac{N}{2}}e^{-\frac{SSE(\beta_{I})}{2\sigma^{2}}}\frac{\beta^{\alpha }}{\Gamma (\alpha )}\sigma^{-2\alpha -2}e^{\frac{\beta }{\sigma^{2}}}d\sigma^{2}=\frac{\beta^{\alpha }}{\Gamma (\alpha )}{\left(\frac{1}{2\pi }\right)}^{\frac{N}{2}}\frac{\Gamma (\alpha +\frac{N}{2})}{{\left(\frac{SSE\left(\beta_{I}\right)+2\beta_{I}}{2}\right)}^{\alpha +\frac{N}{2}}}.$$

Using Bayesian theorem, we can further obtain the posterior distribution of parameter *β*_*I*_ from data **y**,

$$p\left(\beta_{I}|y\right)=\frac{p\left(y|\beta_{I}\right)p\left(\beta_{I}\right)}{\int p\left(y|\beta_{I}\right)p\left(\beta_{I}\right)d\beta_{I}}.$$

Obviously, it is difficult to derive *p*(*β*_*I*_|**y**) directly, we use Markov-Chain Monte-Carlo method to approximate it.

### Metropolis-Hastings algorithm [33]

Suppose *p*(*β*_*I*_|**y**) is the target probability distribution. The process of Metropolis-Hastings algorithm is as follows:

1. Initialization
  1.1 Pick an initial state *β*_*I*0_
  1.2 Set t = 0
2. For each iteration *t*:
  2.1 Generate a random candidate state *β*_*I*1_ according to *f*(*β*_*I*1_|*β*_*I*0_)
  2.2 Calculate the acceptance probability
    $$A\left(\beta_{I0},\beta_{I1}\right)=min\{\frac{p\left(\beta_{I1}|y\right)f\left(\beta_{I0}|\beta_{I1}\right)}{p\left(\beta_{I0}|y\right)f\left(\beta_{I1}|\beta_{I0}\right)},1\}$$
    Substituting Bayesian theorem, we can obtain
    $$A\left(\beta_{I0},\beta_{I1}\right)=min\{\frac{p\left(y|\beta_{I}\right)p\left(\beta_{I}\right)f\left(\beta_{I0}|\beta_{I1}\right)}{p\left(y|\beta_{I}\right)p\left(\beta_{I}\right)f\left(\beta_{I1}|\beta_{I0}\right)},1\}$$
  2.3 Accept or reject
    - Generate a uniform random number *u* ∈ [0, 1]
    - If *u* ≤ *A*(*β*_*I*0_, *β*_*I*1_), then accept the new state and set *β*_*I*(*t*+1)_ = *β*_*I*1_
    - If *u* ≥ *A*(*β*_*I*0_, *β*_*I*1_), then reject the new state and copy the old state forward *β*_*I*(*t*+1)_ = *β*_*I*0_
    - Increment: set *t* = *t* + 1.

In out study, we set the number of iterations *T* = 1, 000, 000 [34]. We suppose the proposal distribution *f*(*β*_*I*(*t*+1)_|*β*_*It*_) and the prior distribution *p*(*β*_*I*_) follow Gamma distribution; the empirical distribution of saved states *β*_*I*0_, *β*_*I*1_, …, *β*_*IT*_ will approach *p*(*β*_*I*_|**y**).

**Fig 10:**
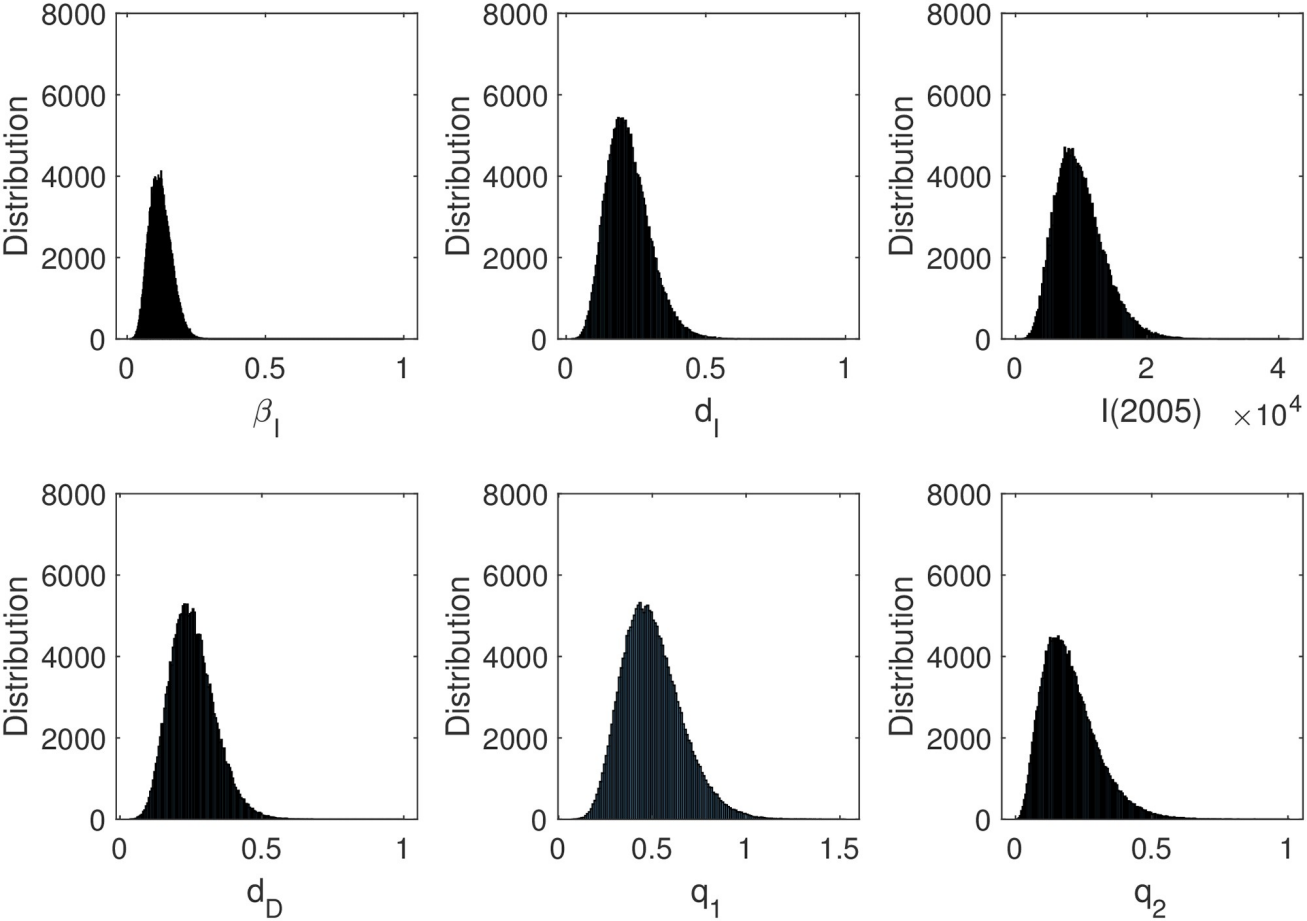
Histogram of parameters. Through Metropolis-Hastings algorithm, the histogram of different parameters is obtained, that is, the distribution of different parameters. The 95% confidence interval of each parameter can be approximately obtained by parameter distribution.

Similarly, we obtain the probability density function of all parameters in Fig 10. Therefore, we get the 95% confidence interval of the above parameters in Table 7.

**Table 7:** Estimated parameters result of MCMC.

Parameter	Description	Mean	95% CI
*d*_*I*_	Death rate of *I*	0.2204	(0.0676,0.3731)
*d*_*D*_	Death rate of *D*	0.2523	(0.0939,0.4107)
*I*(2005)	Initial number of *I*	9987	(2436,17538)
*β* = *cβ*_*I*_	Transmission probability of *I*	0.1208	(0.0411,0.2005)
*q*_1_ = *cβ*_*D*_/*β*_*I*_	Ratio of transmission probability	0.5003	(0.1837,0.8169)
*q*_2_ = *cβ*_*T*_/*β*_*I*_	Ratio of transmission probability	0.2001	(0,0.2005)

## Appendix B. The basic reproduction number

Based on the method in [35, 36], we calculate the basic reproduction number under GACP.

We suppose that $F_{i}$ is the rate of appearance of new infections in compartment *i*; $R_{i}^{+}$ is the rate of transfer of individuals into compartment *i*; $R_{i}^{-}$ is the rate of transfer of individuals out of compartment *i*. Then we have

$$F=(\begin{array}{c} 0\\ \left(1-\eta \right)c\frac{\beta_{I}I+\beta_{D}D+\beta_{T}T+\beta_{G}G}{N}S\\ 0\\ 0\\ 0 \end{array}),$$

and

$$R=R^{-}-R^{+}=(\begin{array}{c} \left(1-\eta \right)c\frac{\beta_{I}I+\beta_{D}D+\beta_{T}T+\beta_{G}G}{N}S-\left(\Lambda -d_{S}S\right)\\ (d_{I}+\alpha )I\\ (d_{D}+\gamma )D-\alpha I\\ (d_{T}+\delta )T-\gamma D\\ d_{G}G-\delta T \end{array}),$$

Based on Lemma 1 in [35], the derivatives DF and DR are partitioned as

$$\begin{array}{cc} DF=[\begin{array}{cc} 0 & J_{1}\\ J_{2} & F \end{array}] & ,DR=[\begin{array}{cc} 0 & J_{3}\\ J_{4} & V \end{array}] \end{array}.$$

Since

$$DF=(\begin{array}{ccccc} 0 & 0 & 0 & 0 & 0\\ \left(1-\eta \right)c\frac{\beta_{I}I+\beta_{D}D+\beta_{T}T+\beta_{G}G}{N} & \left(1-\eta \right)c\frac{S\beta_{I}}{N} & \left(1-\eta \right)c\frac{S\beta_{D}}{N} & \left(1-\eta \right)c\frac{S\beta_{T}}{N} & \left(1-\eta \right)c\frac{S\beta_{G}}{N}\\ 0 & 0 & 0 & 0 & 0\\ 0 & 0 & 0 & 0 & 0\\ 0 & 0 & 0 & 0 & 0 \end{array}),$$

and

$$DR=(\begin{array}{ccccc} \left(1-\eta \right)c\frac{\beta_{I}I+\beta_{D}D+\beta_{T}T+\beta_{G}G}{N}+d_{S} & \left(1-\eta \right)c\frac{S\beta_{I}}{N} & \left(1-\eta \right)c\frac{S\beta_{D}}{N} & \left(1-\eta \right)c\frac{S\beta_{T}}{N} & \left(1-\eta \right)c\frac{S\beta_{G}}{N}\\ 0 & d_{I}+\alpha & 0 & 0 & 0\\ 0 & -\alpha & d_{D}+\gamma & 0 & 0\\ 0 & 0 & -\gamma & d_{T}+\delta & 0\\ 0 & 0 & 0 & -\delta & d_{G} \end{array}),$$

we obtain

$$F=(\begin{array}{cccc} \left(1-\eta \right)\frac{S\beta_{I}}{N} & \left(1-\eta \right)\frac{S\beta_{D}}{N} & \left(1-\eta \right)\frac{S\beta_{T}}{N} & \left(1-\eta \right)\frac{S\beta_{G}}{N}\\ 0 & 0 & 0 & 0\\ 0 & 0 & 0 & 0\\ 0 & 0 & 0 & 0 \end{array}),$$

and

$$V^{-1}=(\begin{array}{cccc} \frac{1}{d_{I}+\alpha } & 0 & 0 & 0\\ \frac{\alpha }{\left(d_{I}+\alpha \right)\left(d_{D}+\gamma \right)} & \frac{1}{d_{D}+\gamma } & 0 & 0\\ \frac{\alpha r}{\left(d_{I}+\alpha \right)\left(d_{T}+\delta \right)\left(d_{D}+\gamma \right)} & \frac{\gamma }{\left(d_{T}+\delta \right)\left(d_{D}+\gamma \right)} & \frac{1}{d_{T}+\delta } & 0\\ \frac{\alpha \delta \gamma }{d_{G}\left(d_{I}+\alpha \right)\left(d_{T}+\delta \right)\left(d_{D}+\gamma \right)} & \frac{\delta \gamma }{d_{G}\left(d_{T}+\delta \right)\left(d_{D}+\gamma \right)} & \frac{\delta }{d_{G}\left(d_{T}+\delta \right)} & \frac{1}{d_{G}} \end{array}),$$

so that

$$R_{C}\left(t\right)=\rho \left(FV^{-1}\right)=\left(1-\eta \left(t\right)\right)\frac{\beta }{d_{I}+\alpha \left(t\right)}(1+\frac{q_{1}\alpha \left(t\right)}{d_{D}+\gamma \left(t\right)}+\frac{q_{2}\alpha \left(t\right)\gamma \left(t\right)}{\left(d_{D}+\gamma \left(t\right)\right)\left(d_{T}+\delta \right)}+\frac{q_{3}\alpha \left(t\right)\gamma \left(t\right)\delta }{\left(d_{D}+\gamma \left(t\right)\right)\left(d_{T}+\delta \right)d_{G}}).$$

where *β* = *cβ*_*I*_, *q*_1_ = *cβ*_*D*_/*β*_*I*_, *q*_2_ = *cβ*_*T*_/*β*_*I*_ and *q*_3_ = *cβ*_*G*_/*β*_*I*_.

## Supporting information

S1 Table HIV/AIDS surveillance data. (PDF)
