# Supplementary material for: Predicting the HIV/AIDS epidemic and measuring the effect of AIDS Conquering Project in Guangxi Zhuang Autonomous Region
Source: PLoS One. 2022 Jul 1;17(7):e0270525. doi: 10.1371/journal.pone.0270525 (PMC9249227; doi:10.1371/journal.pone.0270525)
Supplement: S1 Table — (PDF) [file pone.0270525.s001.pdf]

Supporting Information

HIV/AIDS surveillance data

Year	Total population at the end of a year	Annual HIV-testing individuals	Annual reports	Cumulative reports	Annual alive reports	Annual deaths	Cumulative deaths	Annual enrolment of individuals to ART	Annual individuals under ART	Cumulative individuals under ART	Cumulative deaths under ART	Cumulative individuals who drop out treatment under ART
2005	49250000	91737	6955	17834	17131	600	703	556	421	750	54	19
2010	51590000	4919612	11648	63127	49528	2774	13599	5637	14532	17702	1345	1781
2015	55180000	9484363	9190	106091	69379	2691	36712	9750	48608	62967	5112	8794
2020	57180000	11098389	10671	157352	97293	4266	60059	10961	87284	113750	17871	7306
